# A longitudinal study of cardiac structure and function using echocardiography in patients undergoing peritoneal dialysis

**DOI:** 10.1186/s12882-021-02535-3

**Published:** 2021-10-16

**Authors:** Yunyun Zhu, Difei Zhang, Xiaoxuan Hu, Hui Liu, Yuan Xu, Haijing Hou, Yu Peng, Ying Lu, Xusheng Liu, Fuhua Lu

**Affiliations:** 1 The Second Clinical Medical College, Guangzhou University of Chinese Medicine, 510405 Guangzhou, China; 2 Department of Nephrology, Tongde Hospital of Zhejiang Province, No.234 Gucui Road, Zhejiang Province Hangzhou, China; 3 Department of Nephrology, The Second Affiliated Hospital of Guangzhou, University of Chinese Medicine, No.111 Dade Road, 510120 Guangzhou, China; 4 Department of Nephrology, Guangdong Provincial Hospital of Chinese Medicine, No.111 Dade Road, 510120 Guangzhou, China

**Keywords:** Echocardiogram, Peritoneal dialysis, Cardiovascular disease, End-stage renal disease, All-cause mortality

## Abstract

**Background:**

Peritoneal dialysis (PD) can be associated with abnormal cardiac structure and function and increased mortality risk. Therefore, in this study, we analyzed the cardiac structure and function dynamic changes using echocardiography during the first 2 years of PD therapy. We also assessed its associations with all-cause mortality risk after 2 years of follow-up.

**Methods:**

End-stage renal disease (ESRD) patients that have started PD from 2011 to 2017, and had echocardiography at baseline and years 1 and 2, were included in this study. Echocardiographic parameters were compared between baseline and year 2. Multivariable Cox models were used to estimate the association between echocardiographic parameters changes and all-cause mortality risk.

**Results:**

We finally enrolled 72 PD patients in this study. The mean right ventricular diameter (RVD) increased from baseline (18.31 mm) to year 1 (18.75 mm) and year 2 (19.65 mm). We also observed a significant decrease in cardiac output (CO) between baseline and year 2. Additionally, a slight decrease trend in ejection fraction (EF) was observed. Finally, every 1 % increase in RVD was associated with a 68.2 % higher mortality risk after dialysis (HR, 1.682; 95 % CI, 1.017–2.783).

**Conclusions:**

Our results demonstrated a susceptibility for deteriorated right cardiac structure and function during the first 2 years of PD treatment. Also, higher all-cause mortality risk was observed after 2 years of PD. Altogether, these results highlighted the need for additional focus on regular echocardiographic examinations during long-term PD management.

**Trial registration:**

The PD-CRISC cohort, registered with the Chinese Clinical Trial Registry (ChiCTR1900023565).

## Background

End-stage renal disease (ESRD) patients have higher risks to develop adverse cardiovascular events and even death [1, 2]. Most ESRD patients are maintained on peritoneal dialysis (PD) or hemodialysis (HD). PD has become a well-accepted renal replacement therapy modality for ESRD patients due to its advantages compared to HD, such as greater lifestyle flexibility, lower cost, improved hemodynamic stability, and better residual renal function preservation [3, 4].

However, PD patients can have important cardiac structure and function abnormalities that are associated with adverse clinical outcomes and require frequent subclinical measurements [5, 6]. For example, a previous study revealed that PD patients can have increased mitral annulus velocity and mitral inflow velocity ratio, and diastolic dysfunction indicated by deceleration time [7]. Another study demonstrated that most echocardiographic measurements in PD patients were associated with cardiac valve calcification [8] [including left ventricular mass (LVM) and relative wall thickness (RWT)], cardiovascular events, and mortality [9, 10]. Therefore, cardiac structure and function evaluations in PD patients are crucial in long-term treatments. The first 2 years after PD beginning are especially crucial, due to the peritoneal membrane adaptation to its new environment and fluid overload changes. However, regular echocardiographic parameters monitoring among PD patients seems to be rare.

Therefore, in this study, we analyzed the cardiac structure and function dynamic changes in the first 2 years of PD therapy using echocardiography. We also assessed its associations with all-cause mortality after 2 years of follow-up.

## Methods

### Patients

**Fig. 1:**
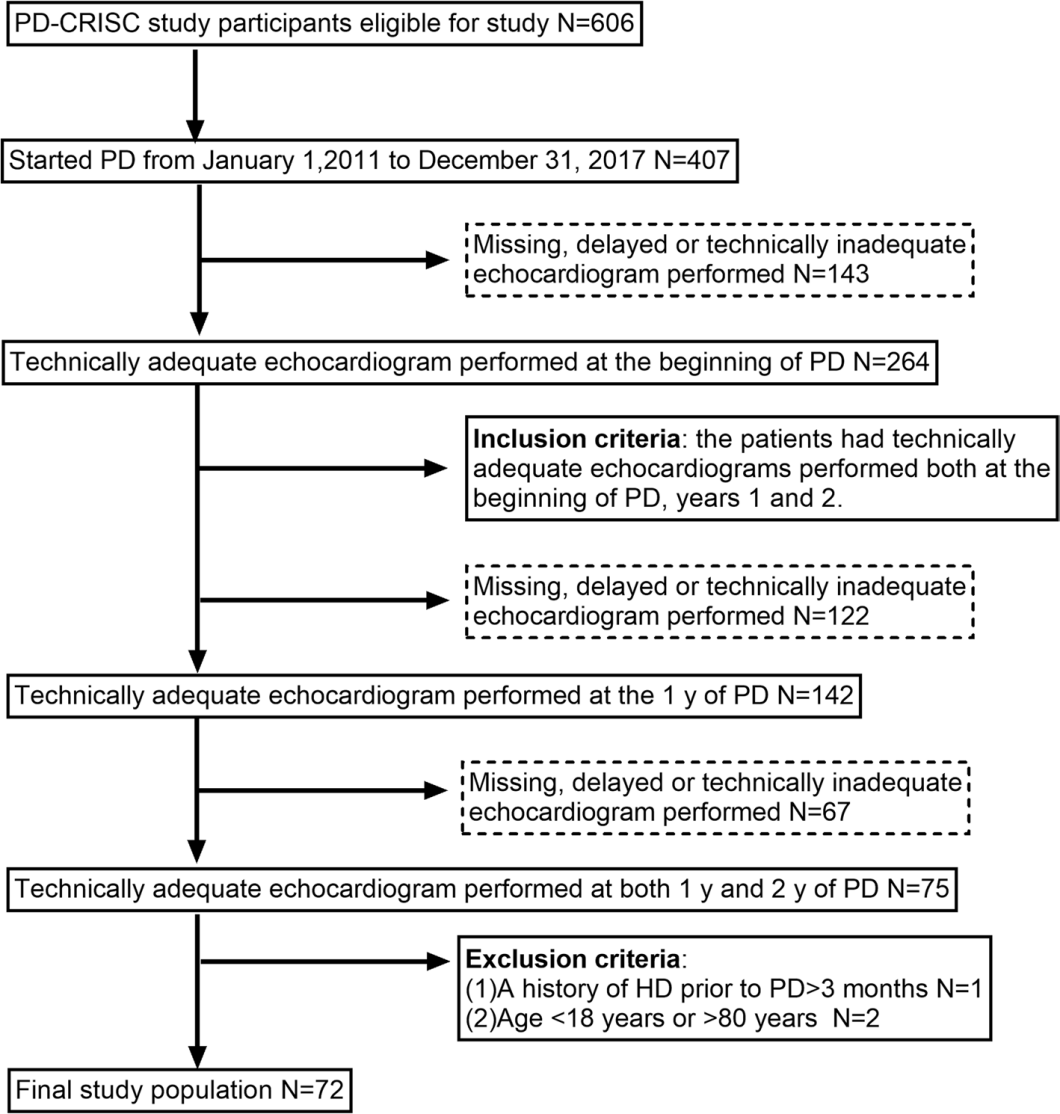
Derivation of the study population

In this study, we enrolled patients from January 1, 2011, to December 31, 2017, with complete follow-ups until January 31, 2019. During this period, 407 patients received PD treatment at the Guangdong Provincial Hospital of Chinese Medicine. The inclusion criteria were: patients with technically adequate echocardiograms performed at PD beginning, and years 1 and 2. The exclusion criteria were: (1) patients who had a history of hemodialysis (HD) before PD – more them 3 months; (2) patients under 18 and over 80 years. Among the 407 patients, 264 had an echocardiogram performed at PD beginning, and 75 also had technically adequate echocardiograms performed at years 1 and 2. Then, we excluded one patient with a history of hemodialysis (HD) before PD, one under 18 years, and one over 80 years. Finally, 72 patients were used for further analyses. Figure 1 represents this study flow chart.

Patients’ data were derived from the PD-CRISC cohort, registered in the Chinese Clinical Trial Registry (ChiCTR1900023565). This study protocol followed the principles established by the Declaration of Helsinki and was approved by the Institutional Ethics Review Boards of Guangdong Provincial Hospital of Chinese Medicine, and The Second Affiliated Hospital of Guangzhou University of Chinese Medicine, Guangzhou, China (ZE2019-123-01).

### Data collection

Patient information was manually collected by medical record revisions and included inpatient charts, outpatient clinic charts, and outpatient dialysis records. Patients also received annual in-person visits to record demographic characteristics, body mass index (BMI), blood pressure level, primary cause, complication, medication use, laboratory test results, weekly renal Kt/V, peritoneal Kt/V, peritoneal Ccr, and renal Ccr, Peritoneal Equilibration Test (PET), and urine output.

### Echocardiographic examinations

Echocardiographic measurements were performed using B-mode, M-mode, and Doppler-mode. All patients were examined in the left lateral decubitus position by a well-trained echocardiographer. Echocardiography was performed according to the American Society of Echocardiography guidelines [11]. All PD patients received 3 echocardiographic examinations: at PD baseline, and years 1 and 2. The following echocardiographic parameters were recorded: left atrial diameter (LAD), left ventricular end-diastolic dimension (LVEDD), left ventricular end-systolic dimension (LVESD), left ventricular posterior wall thickness (LVPWT), interventricular septal thickness (IVST), right ventricular diameter (RVD), right ventricular outflow tract (RVOT), main pulmonary artery (MPA), left ventricular ejection fraction (LVEF), fractional shortening (FS), stroke volume (SV), cardiac output (CO), and pericardial effusion (PE). The LVEF was calculated using standard formulas and data obtained from echocardiography. The E/A (early maximum velocity/late maximum velocity) ratio was also calculated. Finally, the systolic pulmonary artery pressure was estimated based on tricuspid regurgitation.

### Statistical analyses

The patient’s baseline characteristics were summarized and are presented as means ± standard deviations (SD), medians, frequencies, or percentages. We compared PD patients’ laboratory test results over time, and differences in laboratory test results between baseline and year 2. Comparisons were performed using the dependent two-sample Student’s t-test. Echocardiographic parameters from baseline to years 1 and 2 of observation were evaluated for each group using the repeated measures ANOVA, or the χ^2^ test.

Then, we used multivariable Cox models to test associations of all-cause mortality after 2 years of PD treatment with (1) each baseline measure; and (2) changes in each echocardiographic measure from baseline to year 2. We also analyzed associations between baseline variables and echocardiographic changes. Models were adjusted for the corresponding echocardiographic measure at baseline. Multiple linear regression analyses were used for continuous echocardiographic measures and ordinal logistic regressions were used for categorical ones. In the multivariate analysis of the study, baseline variables with *p* values < 0.25 on univariate analysis, and demographic variables, as well as important laboratory indicators, were included for this study. Meanwhile, we also considered the practical reality. A two-tailed *p*-value < 0.05 was considered statistically significant. Statistical analyses were carried out using SPSS version 22.0 for Windows.

## Results

### Patients characteristics’

Patients’ baseline demographic and clinical characteristics are shown in Table 1. The mean age was 52.99 ± 13.84 years, males accounted for 50.9 %, 30.56 % had diabetes mellitus (DM), and 93.06 % had hypertension. The main ESRD primary causes were glomerulonephritis (61.11 %), followed by DM nephropathy (26.39 %), obstructive nephropathy (6.94 %), lupus nephritis (1.4 %), and others (4.17 %).

**Table 1:** Baseline clinical characteristics

Clinical characteristics	Baseline
**Age**(years)	52.99 ± 13.84
**Male** (n, %)	39, 54.2
**BMI** (kg/m^2^)	23.7 ± 3.4
**SBP** (mm Hg)	158.83 ± 20.25
**DBP** (mm Hg)	87.07 ± 15.42
**MAP** (mm Hg)	110.99 ± 15.57
**Primary cause**	
DM nephropathy (n, %)	19, 26.39
Glomerulonephritis (n, %)	44, 61.11
Obstructive nephropathy (n, %)	5, 6.94
Lupus nephritis (n, %)	1, 1.4
Other (n, %)	3, 4.17
**Complication**	
DM (n, %)	22, 30.56
HPT (n, %)	67, 93.06
CVA (n, %)	10, 13.89
CAD (n, %)	6, 8.33
CHF (n, %)	9, 12.50
**Drug**	
ACEI or ARB use	43, 59.72
CCB	59, 81.94
Antidiuretic	24, 33.33
EPO	40, 55.55
Calcium	35, 48.61
**Peritoneal**	
Peritoneal Kt/V weekly	1.36 ± 0.35
Renal Kt/V weekly	0.75 ± 0.46
Peritoneal CCr weekly	36.57 ± 6.71
Renal Ccr weekly	35.91 ± 26.12
PET	1.85 ± 9.68
Urine output(L)	1.02 ± 0.59

Abbreviations: *SBP* systolic blood pressure, *DBP* diastolic blood pressure, *MAP* mean arterial pressure, *BMI *body mass index, *DM* diabetes mellitus, *HPT* Hypertension, *CVA* Cerebral Vascular Accident, *CAD* Cardiovascular disease, *CHF* Congestive heart failure, *ACEI* angiotensin converting enzyme inhibitor, *ARB* angiotensin receptor blocker, *CCB* Calcium Calcium Entry Blockers, *EPO* erythropoietin, *PET* Peritoneal Equilibration Test

### Echocardiographic parameters changes

We further analyzed echocardiographic parameters dynamic changes between baseline, year 1, and year 2 (Table 2). The changes in echocardiographic measures from baseline to year 2 represent " year 2 data” minus “baseline data”.

**Table 2:** The series of echocardiographic parameters

Parameters	Baseline	After 12 months	After 24 months	*P* - value
**Cardiac structure**				
LAD (mm)	36.81 ± 5.68	36.50 ± 6.35	36.89 ± 6.22	0.802
LVESD (mm)	32.76 ± 6.24	32.73 ± 6.98	32.73 ± 7.59	0.974
LVEDD (mm)	51.35 ± 5.64	50.24 ± 6.24	50.50 ± 6.68	0.192
IVST(mm)	12.11 ± 1.87	12.10 ± 1.67	12.33 ± 1.30	0.175
LVPWT(mm)	12.01 ± 1.80	12.03 ± 1.57	12.29 ± 1.19	0.215
RVD(mm)	18.31 ± 2.76	18.75 ± 2.97	19.65 ± 3.00	< 0.001*
RVOT(mm)	25.86 ± 3.74	26.37 ± 3.66	27.7 ± 3.67	0.001*
MAP(mm)	22.08 ± 4.52	22.50 ± 3.60	22.26 ± 2.79	0.885
**Cardiac function**				
FS (%)	36.07 ± 6.54	35.51 ± 7.13	36.07 ± 7.70	0.967
EF (%)	65.01 ± 8.94	64.13 ± 10.50	64.28 ± 10.41	0.867
SV (ml/bit)	81.14 ± 20.70	76.54 ± 17.97	77.35 ± 17.69	0.257
CO (L/min)	6.63 ± 1.85	6.17 ± 1.98	6.13 ± 1.69	0.038*
E/A < 1(n, %)	49, 68.06	53, 73.61	58, 80.56	0.230
PAP > 30mmHg (n, %)	21, 29.17	22, 30.56	25, 34.72	0.757
With PE (n, %)	29, 40.28	15, 20.83	17, 23.61	0.020*

*P* value was estimated from Chi-square test

Abbreviations: *LAD* left atrial diameter, *LVEDD* left ventricular end-diastolic dimension, *LVESD* left ventricular end-systolic dimension, *LVPWT* Left ventricular posterior wall thickness, *IVST* interventricular septal thickness, *RVD* right ventricular diameter, *RVOT* right ventricular outflow tract, *MPA* main pulmonary artery, *FS* Fractional shortening, *EF* left ventricular ejection fraction, *SV *stroke volume, *CO* cardiac output, *E/A* early maximum velocity / late maximum velocity, *PAP* Pulmonary artery pressure, *PE* Pericardial effusion

*P < 0.05

During this period, we detected increases in cardiac structure parameters. The mean RVD increased from baseline (18.31 mm) to year 1 (18.75 mm) and year 2 (19.65 mm) (*p* < 0.001). The median RVOT also increased from 25.86 mm at baseline, to 26.37 mm at year 1, and 27.7 mm at year 2 (*p* = 0.001).

We also detected changes in functional cardiac parameters. A CO significant decrease was detected between baseline and year 2 (*p* = 0.038). Although an EF and SV decrease trend was observed, the difference was not statistically significant. The number of patients with an E/A ratio < 1 increased during the period analyzed, but it was not statistically significant. Finally, a significant decrease in the annual number of pericardial effusion patients was observed (*p* = 0.020).

### Clinical indices changes

We also analyzed patients’ clinical indices at baseline, year 1, and year 2 (Table 3). During the 2-year follow-up, hemoglobin levels improved (*p* < 0.001) but did not reach 11 g. The serum kalium, phosphate, iron, total cholesterol, and uric acid levels presented a declining tendency (*p* < 0.05). On the other hand, carbon dioxide combining power showed an increasing trend (*p* < 0.05). Finally, decreases in creatinine clearance rate and residual urine volume were detected (*p* < 0.05).

**Table 3:** Comparison of patient’s laboratory test results on maintenance peritoneal dialysis over time

Lab test results	Baseline	1 year	2 years	*P*
Hb (g/dL)	79.90 ± 19.24	101.30 ± 24.58	95.01 ± 21.51	< 0.001
K(mmol/l)	4.835 ± 0.09	4.230 ± 0.08	4.24 ± 0.10	< 0.001
Na(mmol/l)	140.10 ± 3.77	139.57 ± 3.41	140.28 ± 2.83	0.217
Cl(mmol/l)	102.70 ± 4.83	98.20 ± 4.13	96.61 ± 11.97	< 0.001
Ca(mmol/l)	1.94 ± 0.28	2.10 ± 0.20	2.19 ± 0.24	< 0.001
P(mmol/l)	1.95 ± 0.61	1.72 ± 0.53	1.88 ± 0.53	0.006
PTH (pg/ml)	460.47 ± 275.10	380.30 ± 220.23	426.74 ± 306.09	0.061
Glucose (mmol/l)	6.43 ± 3.23	6.07 ± 3.14	5.80 ± 3.09	0.359
Scr (umol/l)	999.59 ± 336.53	1001.54 ± 252.83	1091.09 ± 264.00	< 0.001
BUN (mmol/l)	30.30 ± 11.47	21.56 ± 5.75	22.45 ± 7.59	< 0.001
Ccr (ml/min)	4.54 ± 1.74	4.43 ± 2.03	4.01 ± 2.35	0.013
UA (umol/l)	511.81 ± 133.28	428.78 ± 86.30	419.62 ± 90.06	< 0.001
RUV (ml)	1208.91 ± 494.42	919.68 ± 544.44	571.87 ± 470.01	< 0.001
CO_2_CP (mmol/l)	20.64 ± 4.55	25.36 ± 3.37	24.85 ± 3.80	< 0.001
Alb(g/L)	35.43 ± 4.43	32.30 ± 4.68	33.87 ± 5.47	< 0.001
TC (mmol/l)	4.71 ± 1.50	4.24 ± 0.95	4.18 ± 1.09	0.010
TG (mmol/l)	1.53 ± 1.12	1.37 ± 1.18	1.61 ± 1.65	0.146
HDL (mmol/l)	1.17 ± 0.35	1.10 ± 0.34	1.04 ± 0.36	0.048
LDL (mmol/l)	3.04 ± 1.23	2.72 ± 0.85	2.71 ± 1.05	0.083
CK (U/L)	307.03 ± 356.90	255.78 ± 435.43	211.59 ± 353.81	0.006
CK-MB (U/L)	15.13 ± 9.80	16.02 ± 6.77	16.23 ± 7.53	0.830
LDH (U/L)	249.60 ± 70.63	230.65 ± 62.43	226.17 ± 72.03	0.128
Iron(umol/L)	8.29 ± 5.23	7.30 ± 5.35	6.42 ± 3.83	0.006

Abbreviations: *RUV* Residual Urine Volume, *CO*_2_*CP* Carbon Dioxide Combining Power

### Correlations between baseline clinical features and echocardiographic parameters changes

Multivariate models were used to investigate associations between baseline clinical variables and echocardiographic parameters changes (Table 4). Patients with DM were more likely to have an increase in RVD and RA-L from baseline to year 2, and P, K, and BMI were associated with RVOT after 2 years. Older patients were less likely to have a CO decline, and TG was an independent predictor for IVST and LVPWT in year 2. Finally, no significant model was found for LAD, LVEDD, MAP, FS, E/A, SPA, PE.

**Table 4:** Analysis of the association between baseline clinical variables and changes in the echocardiographic parameters from baseline to year 2

Variable	Parameter	B	t	P	R^2^
RVD	DM(with or not)	2.511	3.187	0.002	0.127
RVOT	P	-2.477	-3.303	0.002	0.202
	K	0.247	2.462	0.016	
	BMI	0.279	2.190	0.032	
RVL	DM(with or not)	6.142	2.752	0.008	0.098
RVT	Ccr	1.450	2.377	0.020	0.075
LVESD	Male (VS Female)	2.986	2.066	0.043	0.057
IVST	TG	0.376	2.268	0.026	0.068
LVPWT	TG	0.380	2.431	0.018	0.078
CO	Age	0.040	2.295	0.025	0.070

Multiple linear regression analysis is used for continuous echocardiographic measures; ordinal logistic regression is used for categorical echocardiographic measures. Model is adjusted for age, sex, cardiovascular disease, diabetes, ACE inhibitor or ARB use, BMI, SBP,DBP, Hb, K, P, Ca, Alb, TC, TG, Ccr. No significant model was found in LAD, LVEDD, MAP, FS, E/A, PAP, PE

### Associations between echocardiographic measures changes from baseline to year 2 and all-cause mortality after 2 years of PD treatment

Among the 72 patients, 8 died after a mean PD follow-up time of 3.6 years. The overall death rate was 3.09 per 100 person-years. Other clinical outcomes included two patients that undergone kidney transplantation and five who shift from PD to HD. No significant multivariable model was found for baseline echocardiographic measures and mortality (Table 5).

**Table 5:** Associations of echocardiographic measures at baseline and risk of subsequent all-cause mortality for participants who initiated dialysis

Parameters	Bate	Exp(B)	CI 95 %	*P* - value
**Cardiac structure**				
LAD	0.012	1.012	0.879,1.165	0.872
LVESD	-0.026	0.974	0.835,1.137	0.739
LVEDD	-0.066	0.936	0.802,1.092	0.402
IVST	0.216	1.241	0.675,2.283	0.487
LVPWT	0.269	1.308	0.681,2.512	0.420
RVD	-0.022	0.979	0.660,1.451	0.914
RVOT	0.305	1.356	0.961,1.915	0.083
MAP	0.113	1.120	0.904,1.387	0.302
RA-L	-0.009	0.991	0.890,1.103	0.862
RA-T	-0.063	0.939	0.817,1.079	0.374
**Cardiac function**				
FS	-0.094	0.168	0.797,1.040	0.168
EF	-0.061	0.941	0.860,1.029	0.182
SV	-0.024	0.977	0.939,1.016	0.237
CO	-0.055	0.947	0.558,1.607	0.839
E/A	-0.318	0.727	0.063,8.386	0.798
PAP	1.900	6.686	0.574,77.885	0.129
PE	0.767	2.154	0.369,12.566	0.394

Cox regression model is adjusted for age, sex, cardiovascular disease, ACEI or ARB, diabetes, and BMI from the closest visit to the echocardiographic measures at baseline

*P* value was estimated from Chi-square test

*P < 0.05

Then, we tested the association between each echocardiographic parameter change and all-cause mortality risk after 2 years of PD treatment. RVD increases from baseline to year 2 were significantly associated with greater mortality risk after 2 years on PD (Table 6). Every 1 % increase in RVD was associated with a 68.2 % higher mortality risk after 2 years on PD (HR, 1.682; 95 % CI, 1.017–2.783).

**Table 6:** Associations of changes in echocardiographic measures from baseline to year 2 and risk of all-cause mortality

Parameters	Bate	Exp(B)	CI 95 %	*P* - value
**Cardiac structure**				
LAD	0.006	1.006	0.881,1.148	0.930
LVESD	0.065	1.067	0.902,1.261	0.449
LVEDD	0.041	1.042	0.879,1.235	0.634
IVST	-0.033	0.968	0.414,2.264	0.940
LVPWT	0.326	1.386	0.570,3.368	0.471
RVD	0.520	1.682	1.017,2.783	0.043*
RVOT	-0.121	0.886	0.700,1.122	0.314
MAP	-0.036	0.965	0.782,1.190	0.737
RA-L	0.037	1.038	0.931,1.157	0.504
RA-T	-0.009	0.991	0.909,1.081	0.842
**Cardiac function**				
FS	0.108	1.114	0.989,1.254	0.075
EF	-0.077	0.925	0.796,1.075	0.312
SV	0.015	1.015	0.974,1.058	0.472
CO	0.395	1.484	0.762,2.888	0.246
E/A	-0.991	0.371	0.033,4.181	0.423
PAP	0.133	1.142	0.175,7.459	0.890
PE	-1.783	0.168	0.008,3.712	0.259

Cox regression model is adjusted for age, sex, cardiovascular disease, ACEI or ARB, diabetes and BMI from the closest visit to the echocardiographic measures at baseline

*P* value was estimated from Chi-square test

*P < 0.05

## Discussion

In the present study, we found that right ventricle structural changes occurred before left ones during the first 2 years of PD. Although the left heart was stable, its functions showed a downward trend. We also found that an increase in RVD from baseline to year 2 was significantly associated with higher mortality risk after 2 years on PD.

Echocardiography is an established technique to estimate cardiovascular complications risks in ESRD patients. It is also widely available and recommended for ESRD patients’ diagnosis and treatment guidance. A previous study had recommended that echocardiography should be repeated at 1.5-year intervals for dialysis patients. However, most patients in that study received HD [12]. Additionally, clinically meaningful echocardiographic parameters are not fixed and can progress, or regress, even in post-dialysis [6, 13]. Therefore, cardiac echocardiographic evaluations in PD patients are crucial during long-term management.

Previous studies on long-term observation of echocardiographic parameters of PD patients mainly focused on the left heart. A recent study suggested that long-term PD patients maintained stable left ventricular (LV) structure and systolic functions, but the cardiac diastolic function declined over time [14]. Another study reported a reduction in LVMI and LA size on PD patients followed over 24 months [15]. Also, a previous study showed that LV hypertrophy was observed in 78 % of the patients at baseline and 60 % after 18 months of PD treatment. LV systolic and diastolic functions were significantly better after 18 months of PD treatment [16].

On the other hand, monitoring the right heart during PD treatment is rarely involved. RVD is one of the most important echocardiographic indicators of RV structure and function. It has been hypothesized that RVD is an indicator for all-cause mortality prediction in heart failure with preserved ejection fraction (EF) [17]. This is consistent with our study since we find an RVD increment at years 1 and 2, which was associated with higher post-dialysis mortality risk. This increase can potentially account for a right-side cardiac loading increase, mostly caused by pulmonary hypertension. We observed pulmonary hypertension in about a third of our patients (Table 2), that had no pulmonary or immune-related diseases. Although the difference did not reach statistical significance, a decreasing trend was observed during the period of evaluation. Similar findings have been reported elsewhere [18, 19]. For example, pulmonary hypertension was found in 20.69 % in pre-dialysis ESRD patients, 14.7 % in PD patients, and 16.7 % in HD patients. It has been suggested that uremia would play a major role in patients’ PH pathogenesis [19]. We hypothesized that ESRD itself would lead to pulmonary hypertension, which is worthy of further study. Moreover, the residual renal function (RRF), which associated with the fluid status of PD patients, decline can be another RVD change cause. RRF preservation is important to conserve cardiac performance, resulting in an improvement in PD patients’ clinical outcomes [20]. In a previous study [21], PD patients presented RRF every six weeks for 2 years. After echocardiography, the right ventricular systolic pressure was associated with faster RRF declines. In our patients, RRF (residual urine volume and creatinine clearance) declined within 2 years, which can be one of the RVD change causes.

Conventionally, EF - a global cardiac function marker - is often used as a systolic function measure. A previous study found that the EF presented significant improvement after 12 months of PD [8]. However, another study showed that PD patients’ EF did not show significant changes during 3 years of follow-up [14], consistent with our findings. We believe that the hemoglobin, plasma albumin, and blood electrolyte improvements in patients during the first 2 years of PD make their cardiac function relatively stable. It should be noted that, although the EF was not statistically significant, there was an overall decrease trend. First, during our 2-year follow-up, hemoglobin levels in this population improved, but still did not reach 11 g. Also, a slight decrease in plasma albumin was detected, which could affect cardiac outcomes. Second, uremia was independently associated with worse cardiac mechanics [14] and requires close monitoring and attention in clinical practice.

DM is considered a high cardiovascular disease risk. Previously, gender (male), DM history, and BMI were used to predict cardiac structure and function variations in PD patients [16, 22, 23]. In our study, we also found that male PD patients with diabetes were more likely to have RVD changes after 2 years. Although the KIDGO guidelines [24] recommend that echocardiography should be performed every 3 years for dialysis patients, we believe that the interval should be shortened for these PD patients to detect cardiac problems earlier and adjust medications.

Our study strengths include the dynamic collection of complete echocardiographic data in the first 2 years of PD therapy to investigate their changes. We also assessed the relationship between cardiac changes and all-cause mortality after the long-term follow-up. Finally, we simultaneously analyzed the association of echocardiographic changes with clinical indices.

This study also has some limitations. It was a single-center, retrospective, observational study with a small sample size and short follow-up time. Although 407 patients received PD during our study period, only 264 had echocardiography at PD beginning. Our objective was to analyze cardiac structure and function dynamic changes. The echocardiography was required at three points: baseline, and years 1 and 2. However, the previous K/DOQI clinical practice guidelines recommendation is that dialysis patients should have an echocardiography every three years. Therefore, in our study, the cases finally included comprehended only 27.3 % (72/264) of the first included. Additionally, although retrospective studies have shown selection bias, we can not deny that this study’s cohort reflects the inadequacy of our PD center’s regular evaluation of patients’ echocardiography. Thus, we should pay more attention and improve that in the future.

## Conclusions

Overall, this study demonstrated the dynamic changes in cardiac structure and function in PD patients over time. Our findings highlighted the susceptibility for deteriorated right cardiac structure and function in the first 2 years of PD treatment. Also, we detected a higher all-cause mortality risk beyond 2 years after PD. These results can be used to increase the focus on regular echocardiographic examinations during long-term PD management.

## Data Availability

The datasets used and/or analyzed during the current study are available from the corresponding author on reasonable request..

## Abbreviations

ESRD: End-stage renal disease
PD: Peritoneal dialysis
HD: Hemodialysis
LAD: Left atrial diameter
LVEDD: Left ventricular end-diastolic dimension
LVESD: Left ventricular end-systolic dimension
LVPWT: Left ventricular posterior wall thickness
IVST: Interventricular septal thickness
RVD: Right ventricular diameter
RVOT: Right ventricular outflow tract
MPA: Main pulmonary artery
FS: Fractional shortening
EF: Left ventricular ejection fraction
SV: Stroke volume
CO: Cardiac output
E/A: Early maximum velocity / late maximum velocity
PAP: Pulmonary artery pressure
PE: Pericardial effusion
